# Genome-wide Association Study Identifies Loci for the Polled Phenotype in Yak

**DOI:** 10.1371/journal.pone.0158642

**Published:** 2016-07-07

**Authors:** Chunnian Liang, Lizhong Wang, Xiaoyun Wu, Kun Wang, Xuezhi Ding, Mingcheng Wang, Min Chu, Xiuyue Xie, Qiang Qiu, Ping Yan

**Affiliations:** 1 Key Laboratory of Yak Breeding Engineering Gansu Province, Lanzhou Institute of Husbandry and Pharmaceutical Sciences, Chinese Academy of Agricultural Science, Lanzhou, China; 2 State Key Laboratory of Grassland Agro-ecosystem, College of Life Science, Lanzhou University, Lanzhou, China; University of Lleida, SPAIN

## Abstract

The absence of horns, known as the polled phenotype, is an economically important trait in modern yak husbandry, but the genomic structure and genetic basis of this phenotype have yet to be discovered. Here, we conducted a genome-wide association study with a panel of 10 horned and 10 polled yaks using whole genome sequencing. We mapped the *POLLED* locus to a 200-kb interval, which comprises three protein-coding genes. Further characterization of the candidate region showed recent artificial selection signals resulting from the breeding process. We suggest that expressional variations rather than structural variations in protein probably contribute to the polled phenotype. Our results not only represent the first and important step in establishing the genomic structure of the polled region in yak, but also add to our understanding of the polled trait in bovid species.

## Introduction

The yak (*Bos grunniens*) is endemic to the Qinghai-Tibet Plateau (QTP), the largest and harshest highland in the world [1]. More than 14 million domestic yaks are currently distributed across the QTP, providing the food, shelter, fuel and transport that enable nomadic Tibetans and other pastoralists to survive in this harsh climate; indeed, the yak has become an iconic symbol of Tibet [1, 2]. They are also strongly integrated into Tibetans’ socio-cultural life. Due to its important position in Tibetan daily life, yak production and its related products are the cornerstone of Tibetan animal husbandry [3, 4]. In addition, yak live on unpolluted highland pasture where they produce green, nutritional and healthy products, much valued by modern communities [3, 4].

**Fig 1:**
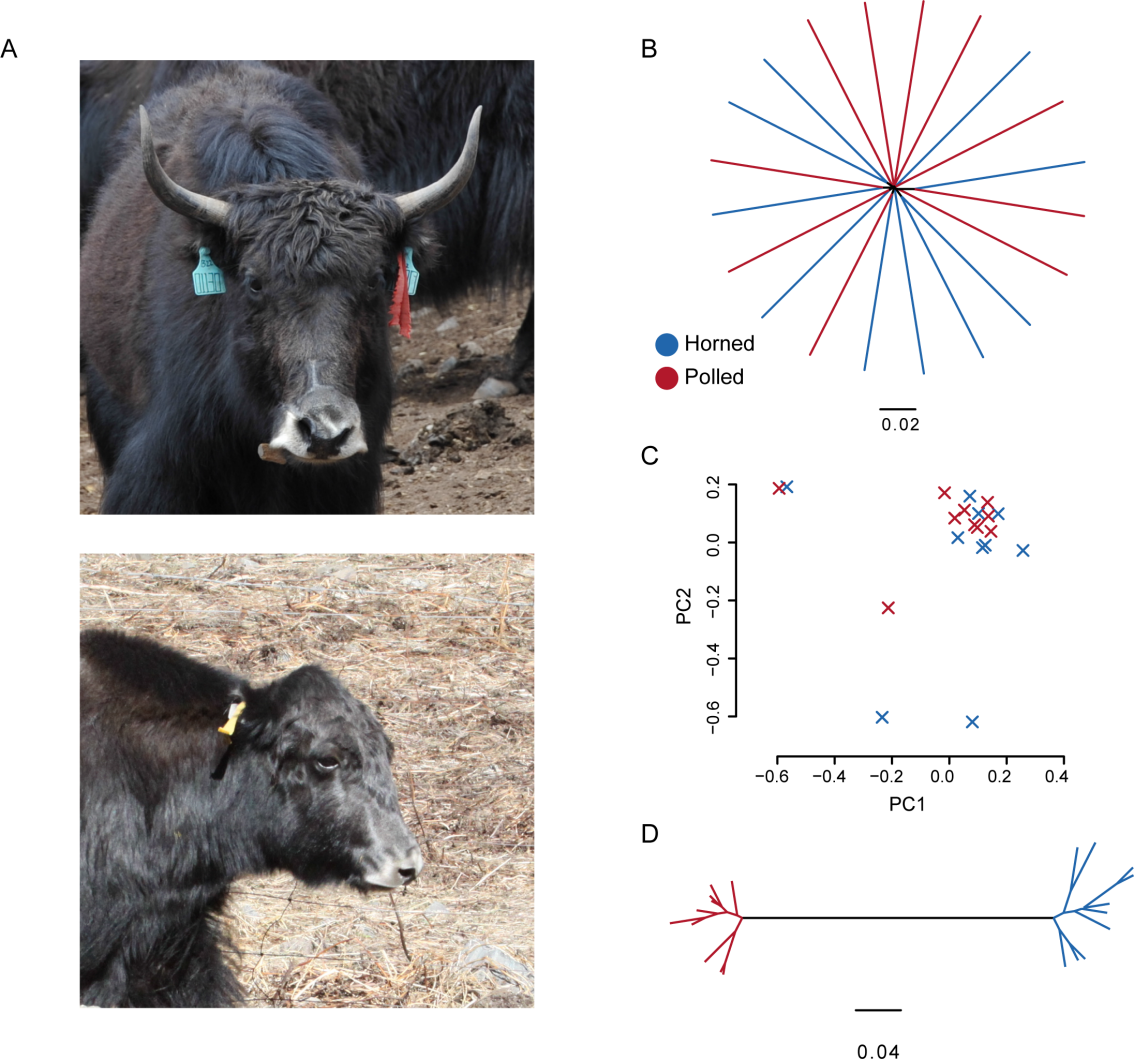
Phylogenetic and population structure of horned and polled yaks. (**A**) Photos of horned (above) and polled (below) yak herds, taken by Chunnian Liang. (**B**) A neighbor-joining phylogenetic tree constructed using whole-genome SNP data. The scale bar represents level of similarity. Horned (blue) and polled (red) samples are indicated. (**C**) Principal component (PC) analysis plots of the first two components. (**D**) A neighbor-joining phylogenetic tree constructed using SNP data for the GWAS region.

The bovine polled phenotype, the highly desired and favorable trait in modern husbandry systems, has huge practical importance for breeders and is of special interest to geneticists [5, 6] (Fig 1A shows a horned and a polled yak). Yak horns are a major cause of injuries, particularly in feedlots and during transport [7]. Nowadays, commercial beef yaks are confined to barns and fenced-in enclosures such as pastures or corrals. More hornless yak can be accommodated in the same space compared to yak with horns, and the trait would reduce economic losses due to injuries to both humans and animals under these conditions [7]. Although dehorning at a young age is routinely practiced in horned breeds of yak, this method does not eradicate the problem and there are associated animal welfare concerns. Considering an autosomal dominant mode of inheritance for the polled trait, the approach also limits the ability to discriminate between heterozygous and homozygous polled animals [8, 9]. Thus, creating polled genetic markers to identify homozygous/heterozygous yak and breeding genetically polled yak based on non-invasive and high welfare methods is a promising alternative [7, 9], which would be valuable to modern yak husbandry in high altitude harsh environments [10]. In addition, identification of genes and causal variations associated with the polled phenotype will contribute to our knowledge and understanding of the molecular mechanisms that underlie horn differentiation and development in bovine species.

In cattle (*Bos taurus*), the *POLLED* locus has previously been mapped on the proximal end of bovine chromosome 1 (BTA1) [11]. More recent efforts to refine the polled locus and detect candidate causal mutations have included seeking additional microsatellite markers [12–14], BAC-based physical mapping [15], high-density SNP genotyping [16–19], targeted capture sequencing [17, 20, 21] and whole genome resequencing [16, 19]. Recently, at least two different alleles for polledness were reported in cattle, identifying an 80-kb duplication (BTA1: 1,909,352–1,989,480 bp) in Friesian original breeds (P_F_ allele) and a duplication of 212-bp (BTA1: 1,705,834–1,706,045 bp) in place of a 10-bp deletion (1,706,051–1,706,060 bp) in Celtic original breeds (P_C_ allele, a 202-bp insertion–deletion, InDel), respectively, suggesting the existence of allelic heterogeneity at the *POLLED* locus [17, 21, 22]. In addition, other sporadic mutations associated with the horn-like scurs phenotype have been described [23, 24]. Intriguingly, none of these mutations was located in known coding or regulatory regions [13, 16]. One plausible reason is that different alleles have been selected in different geographic regions or breeds, and world-wide and across-breed breeding using limited founders and artificial insemination have led to the complex inheritance pattern of the horn trait in different breeds, thus adding to the complexity of understanding the molecular basis of polledness [17]. Hence, simultaneous research in different Bovidae species needs to be undertaken to provide extra information [7, 19].

In the current breeding stage, polled yaks are developed deliberately by crossing polled cows (PP or Pp) with horned bulls (pp) by artificial insemination (S1 Fig) at the Datong Yak Breeding Farm of Qinghai Province, providing an ideal system to study the genomic structure and genetic basis of this phenotype [10]. Although our previous analysis based on an a priori candidate gene set detected associated signals, the actual location of the *POLLED* locus has so far not been confirmed as BTA1 in yak [10]. In the present study, we describe our efforts to determine the polled trait associated genome regions in yak based on whole genome sequencing; these were carried out independently from any recently published studies.

## Results and Discussion

### Genetic variants and population structure

We sequenced 10 horned and 10 polled yaks to an average depth (raw data) of 11.2× using an Illumina Hiseq2000 instrument, resulting in a total of 6.04 billion reads comprising approximately 595Gb of sequencing data. Using BWA-MEM software [25], reads were aligned to the *B*. *grunniens* reference genome with an average alignment rate of 91.20%, covering 99.26% of the genome [26] (S1 Table). After SNP calling using SAMtools [27], filtering the potential PCR duplicates, removing SNPs with potential errors and correcting the misalignments around InDels (details in Materials and Methods), approximately 8.4 million high quality SNPs were retained.

To examine the phylogenetic relationship between horned and polled yaks, we constructed a neighbor-joining tree based on our high-quality SNPs. The horned and polled yaks formed a mixed clade (Fig 1B), indicating that pairwise distances between horned and polled yaks were not larger than those within each population. We also performed principle component analysis (PCA, Fig 1C and S2 Table) and population structure analysis (S2 Fig) based on the genotype data. Taken together, all of these results indicate no population genetic structure correlated with the horned/polled phenotypes, consistent with the relatively short time of breeding polled yaks. More importantly, the indistinguishable genomic background suggests that genome-wide association studies should enable high-resolution mapping of genomic regions associated with the horned/polled phenotype.

### Identification of genome regions associated with the polled phenotype

**Fig 2:**
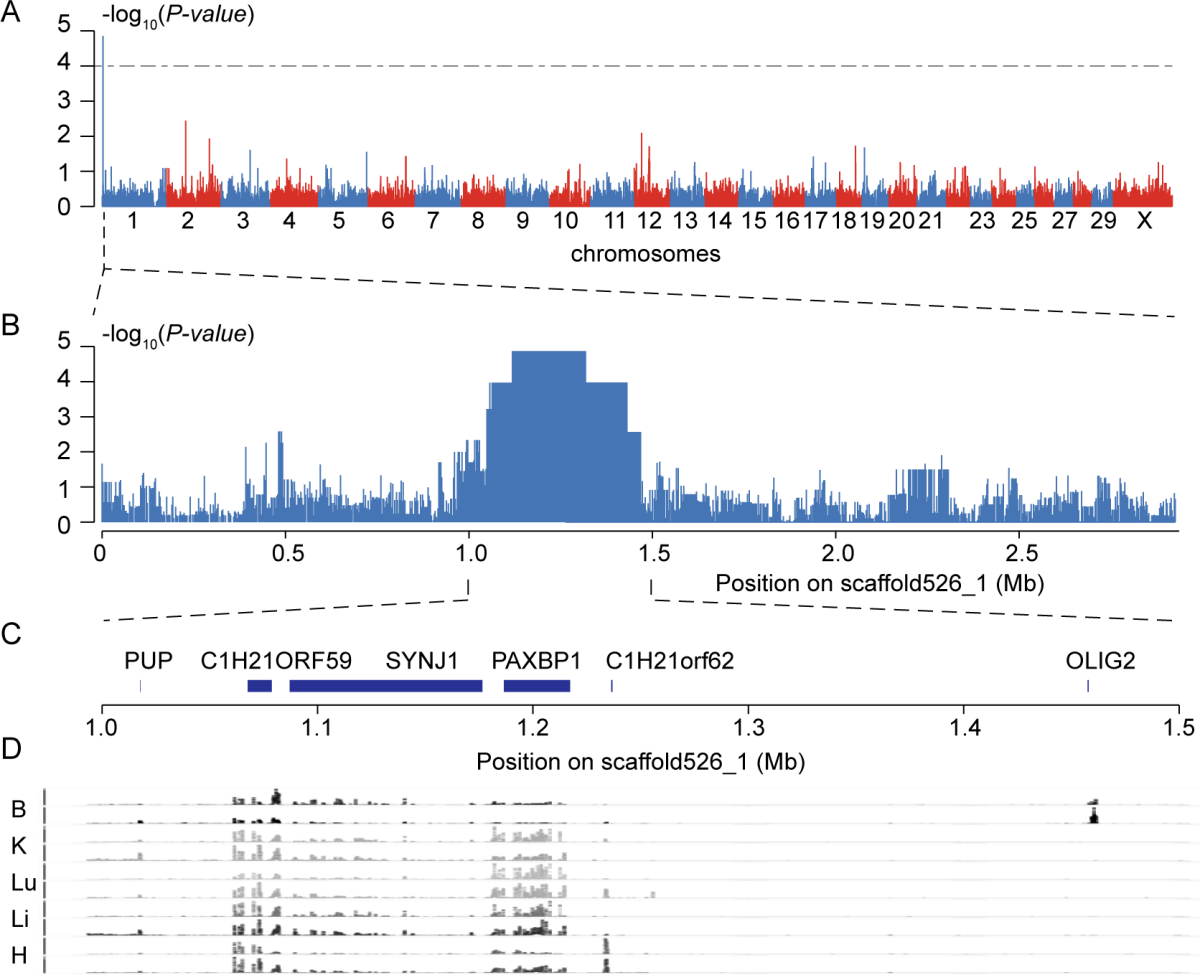
Associated mapping of the polled phenotype. (**A**) Genome-wide *P* values (y axis) are plotted along the genome (**B**) and magnification of scaffold526_1. (**C**) All genes around the candidate GWAS region. (**D**) diagram of read depths (X axes) of RNA-seq data mapping of five different tissues: brain (B), kidney (K), lung (Lu), liver (Li) and heart (H), each with two replicates (Y axes).

Taking advantage of the yak population with no population stratification, we performed a genome-wide association study (GWAS) analysis between 10 horned and 10 polled animals using the Dominant model with PLINK [28]. This autosomal dominant Mendelian trait was mapped to a 200 kb interval between positions 1,122,103 and 1,322,666 bp on scaffold526_1 (*P*-values<0.0001, which means that the probability of obtaining these frequencies by chance is very low, <0.01%) (Fig 2A and 2B, S3 Fig). Despite the small sample size and the consequent relatively limited statistical power [29], this exclusive signal reached genome-wide significance in the GWAS analyses and appeared to align within the polled locus of BTA1 mapped in previous studies of cattle [11–19, 21]. Further, we found that the horned and polled individuals clustered into two genetically distinct groups in a phylogenetic tree based on this region (Fig 1D). In contrast, there was no significant differentiation between these groups when the tree was constructed for the whole genome (Fig 1B). Moreover, most of these loci with *P*-values<0.0001 (95%, 567 of 596) are entirely heterozygous (Pp) in polled animals, coinciding with the breeding practice of crossing polled cows (PP or Pp) with horned bulls (pp) by artificial insemination. Although all previous studies identified different polled mutations in different cattle breeds, all the clues lead to the same position on BTA1 [11–19, 21]; we therefore believe that the horned/polled trait in cattle and yak may share the same ontogenetic mechanism.

The region defined as the GWAS loci of the polled phenotype contained 3 protein-coding genes: *SYNJ1*, *PAXBP1* and *C1H21orf62* (Fig 2C). *SYNJ1* encodes synaptojanin 1, a key neural protein highly expressed in nerve terminals with essential roles in the regulation of synaptic vesicles in conventional synapses and hair cells [30, 31]. Recently, a histological analysis revealed that nervous tissue and hair follicle development have different features in horn buds and polled frontal skin during the development of the horn buds of bovine fetuses, implying that *SYNJ1* maybe have an important role in horn differentiation [32]. *PAXBP1* is an essential binding protein that regulates the proliferation of muscle precursor cells, which in turn, are involved in the development of normal craniofacial features and spine morphogenesis [33]. *C1H21orf62* is an uncharacterized protein. Since previous transcript profiling analyses of polled and horned tissues from cattle, using the Agilent 44 k bovine array, failed to find differential expression in any of the genes located in the *POLLED* locus [34], we sought to re-annotate the novel protein-coding genes in this region by mapping large-scale RNA-seq data for five tissues (brain, kidney, lung, liver and heart) from a previous study [35] of domestic yak. We were, however, unable to find any new gene or open reading frame (Fig 2D, details in Materials and Methods).

Previous genetic and genomic research has proposed two structural variants (P_F_ and P_C_ alleles) associated with the polled phenotype based on larger scale GWAS results in many cattle breeds [17]. We therefore examined whether these two structural variants exist in the yak genome based on our whole genome sequencing data (> 10×), which should ensure quality and accuracy in the detection of structural variants [36]. Our results indicated that the yak genome does not include these two cattle candidate structural variants (S4 Fig and S3 Table), contrasting with previous studies indicating that there is extensive allelic heterogeneity of the polled trait in highly mobile bovid species. We further analyzed other structural variants from this associated region and could not identify any duplications or InDels associated with the polled trait in yak (S3 Table). Due to the small sample size, short breeding history and absence of homozygous polled (PP) individuals, our results cannot be used to refine the candidate genomic region nor to detect causal variants (causal SNPs or structural variants) in yak at present. However, we are convinced that a future study based on more samples with detailed pedigree information will narrow down the candidate region of this trait [37], and this highly confidential region should be the target of focused studies to establish the functional significance of this key trait in domestic yak.

### Characterization of the polled interval

**Fig 3:**
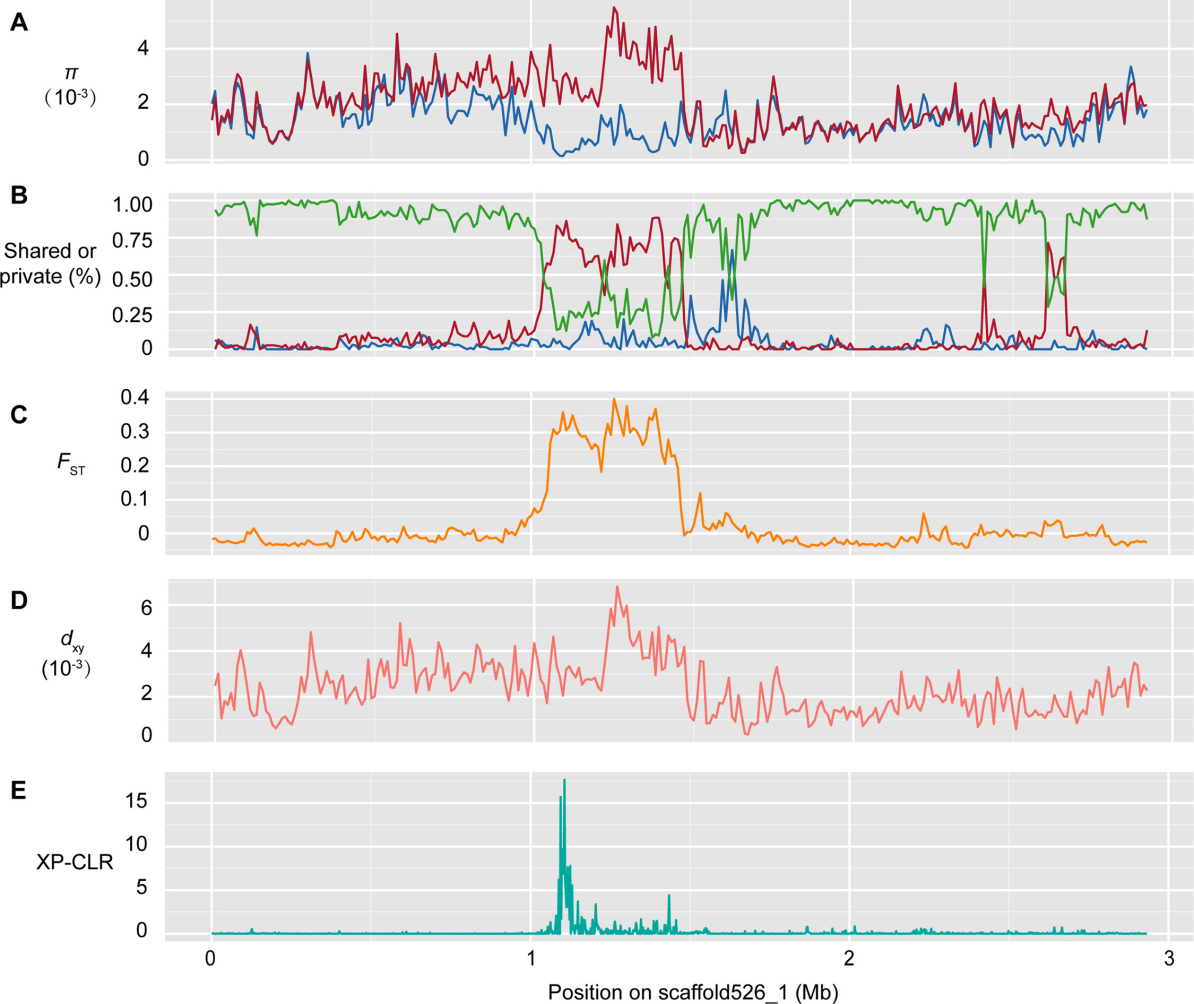
Distribution of population genomic parameters along scaffold526_1. The plots show: (**A**) the nucleotide diversity (π, blue for horned and red for polled yaks) for each population; (**B**) the proportion of shared polymorphisms among sites that are polymorphic in at least one population (green), the proportion of private polymorphisms among sites that are polymorphic within populations (blue for horned and red for polled yaks), private and shared polymorphisms shown in the same panel; (**C**) *F*_ST_; (**D**) *d*_xy_; and (**E**) XP-CLR of scaffold526_1.

**Fig 4:**
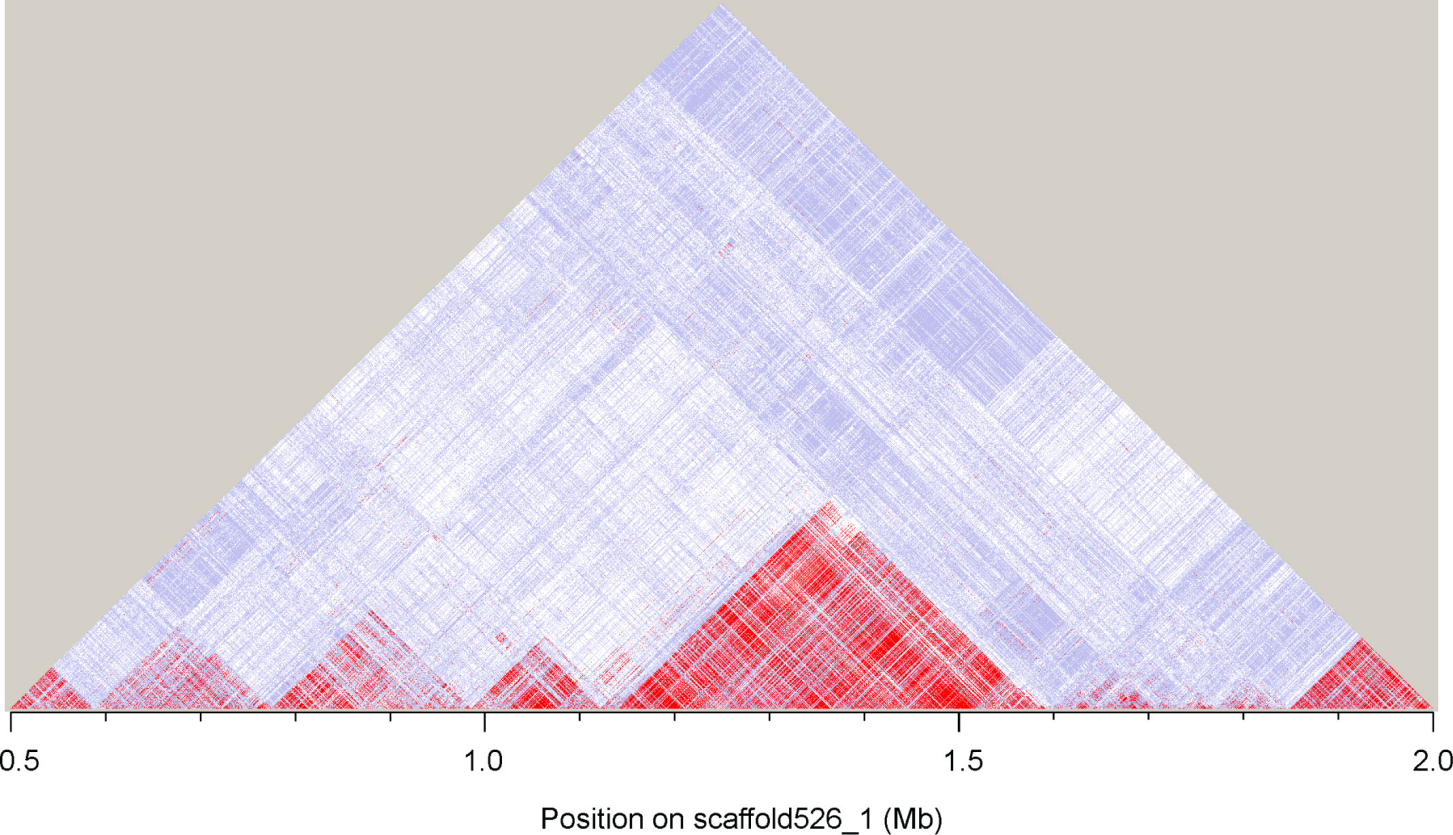
Haplotype block at linkage disequilibrium along the scaffold526_1.

Considering the breeding practice developed by crossing polled cows (PP or Pp) with horned bulls (pp) (Schematic shown in S1 Fig), the candidate region identified could be expected to exhibit specific signatures of recent artificial selection in the polled population, including a high proportion of heterozygous, significantly differentiated nucleotide diversity levels and long-range haplotype homozygosity [38]. Based on these principles, we examined five different parameters to evaluate detailed genetic polymorphism and differentiation between horned and polled yaks: nucleotide diversity (π), the proportion of shared and private SNPs, *F*_ST_ (population-differentiation statistic), *d*_xy_ (mean pairwise comparisons of the nucleotide difference between groups) and the linkage disequilibrium (LD). Population-specific estimates of π showed that the level of nucleotide diversity is higher in polled yaks than in horned yaks in the 200 kb GWAS region (Fig 3A), although a similar level of nucleotide diversity was observed in the rest of genome (mean pairwise nucleotide diversity of π_horned_: 0.00139±0.00081; π_polled_: 0.00136±0.00077, S5 Fig). In addition, this region showed an elevated proportion of private SNPs in polled yaks and a reduced level of shared SNPs between horned and polled yaks (Fig 3B), which also accord with the breeding approach. Furthermore, we found that the GWAS region implicated in the polled phenotype showed striking genetic differentiation between the horned and polled individuals in the *F*_ST_ analysis (Fig 3C, with a mean *F*_ST_ of only 0.0006, S6 Fig). The mean pairwise nucleotide difference between-group comparisons (*d*_xy_) showed a divergence peak (more than 0.006) compared with the flanking regions (Fig 3D, with whole genome level of 0.0013±0.0006). Linkage analysis for this scaffold also revealed a higher linkage disequilibrium (LD) value with one haplotype block of 450 kb (1.14~1.59Mb, Fig 4), which was probably defined by the causal allele and its linked neighbor variants. Taken together, these results indicate that this candidate genomic region tends to be highly diverged and exhibits clear signals of selection. As a complementary approach, we used a likelihood method (the cross-population composite likelihood ratio, XP-CLR [39]) to scan for extreme allele frequency differentiation over extended linked regions and found this region to have elevated XP-CLR values (Fig 3E), which means that the polled trait was affected by selection during breeding activities.

### Potential genetic basis and future directions

Despite the fact that the *POLLED* locus has been easily mapped, fine characterization of this locus and a definitive description of the molecular basis of horn ontogenesis has proved more difficult than expected [17, 22]. One important reason is that complex genomic structural variations and a possible regulatory effect rather than nonsynonymous variations in protein sequence are the major contributors to the differentiation of the horned/polled trait [16, 17, 19, 21]. A recent study reported the success of production of hornless dairy cattle using genome editing technology involving introgressing the Celtic original candidate *POLLED* allele (202-bp insertion–deletion), but the potential functional effect of this sequence variant remains unknown [22]. To date, none of the causative variants have been located in known coding or regulatory regions and no genes with a high probable impact have been identified in cattle [13, 16]. To test this result in yak, we scanned the SNPs within the coding regions of the *POLLED* locus and were unable to identify any nonsynonymous substitutions, consistent with the situation previously observed in cattle. In addition, differential expression studies among horned and polled cattle failed to reveal differences in gene expression located in the *POLLED* locus, but several genes outside this region showed a high level of expression divergence [19].

By combining our results with those of earlier studies, we suggest that unknown regulatory sequences and cis-regulation elements may reside in the *POLLED* locus, thus influencing horn development. Furthermore, the horned/polled trait is developmentally-related, and such traits are likely to be involved in the complex interaction of many genes [16, 19, 35]. This indicates that a high level of genetic heterogeneity is expected and that different species or breeds may have developed this phenotype as a result of different genomic strategies [17]. For example, sequence changes in the *POLLED* locus may affect the transcription factor and noncoding RNA binding, coordination of histone modifications and other chromatin remodeling activities, which can lead to transcription changes to horn-related genes in the *POLLED* locus and other regions of the genome [40]. Therefore, we must emphasize the important role of gene regulation in horn development. Future studies should focus on identification of novel regulated elements in the *POLLED* locus and involve an examination of detailed expression patterns across different horn developmental stages, which should reveal the precise mechanism responsible for the polled trait.

## Conclusions

The importance of breeding polled yak has grown considerably due to animal welfare issues and the needs of modern yak husbandry. Herein, we report the first genome-wide association study of the *POLLED* phenotype in which we identified a 200-kb genomic region responsible for this economically important trait in yak. However, we need to point out that the candidate region was inevitably large because of the small sample sizes, and our current data were insufficient to detect causal variants. Further research based on larger sample sizes will be necessary to obtain more reliable estimates and refine the genomic loci that contribute to this trait. We found that this region was under artificial selection and the characterizations of the *POLLED* locus were concordant with the breeding process. Combined with previous results in cattle, we further suggest that expressional variations other than structural variations in protein are the major causes of the polled phenotype. The results of our study represent a critical advance towards the delimitation of a genomic region for further functional study and provide new insights into the genetic basis of the polled trait in yak and other bovine species.

## Materials and Methods

### Sample collection and sequencing

We randomly selected 10 horned and 10 polled individuals (S1 Table) of Datong yaks from a large herd (n > 2000, from the Datong Yak Breeding Farm of Qinghai Province (37°15'35.6"N, 101°22'24.0"E). For each yak, genomic DNA was extracted from blood samples using a standard Phenol/Chloroform method. The quality and integrity of the extracted DNA was controlled by the A260/A280 ratio and agarose gel electrophoresis. Paired-end sequencing libraries with an insert size of 500 bp were constructed according to the Illumina manufacturer’s instructions, for sequencing on the Hiseq 2000 platform, and paired-end reads were generated. Sequencing and base calling were performed according to the standard Illumina protocols. All individuals were sequenced to an average raw read sequencing depth of 11.2× assuming a genome size of 2.66 Gb. All experimental protocols were approved by ethical committees of the Datong Yak Breeding Farm of Qinghai Province.

### Sequence quality checking and mapping

We performed a per-base sequence quality checks [41] and low quality reads of the following types were filtered out: (i) Reads with ≥10% unidentified nucleotides (N); (ii) Reads for which more than 65% of the read length had a phred quality score ≤7; (iii) Reads with more than 10 bp aligned to the adapter, allowing 2 bp mismatches; and (iv) duplicate reads. Reads were also trimmed if they had three consecutive base pairs with a phred quality score of 13 or below, and discarded if they were shorter than 45 bp.

The pair-end sequence reads were mapped to the *B*. *grunniens* reference genome using BWA-MEM [25] (0.7.10-r789) with default parameters. The *picard* software (http://broadinstitute.github.io/picard/, version 1.92) was subsequently used to assign read group information containing library, lane, and sample identity. Duplicated reads were filtered and index files were built for reference as well as bam files using SAMtools (0.1.19). The Genome Analysis Toolkit (GATK, version 2.6-4-g3e5ff60) [42] was used to perform local realignment of reads to enhance the alignments in the vicinity of InDel polymorphisms. Realignment was performed with GATK in two steps. The first step used the RealignerTargetCreator to identify regions where realignment was needed, and the second step used IndelRealigner to realign the regions found in the first step, generating a realigned mapping file for each individual. The overall mapping rate of reads to the reference genome was 91.20%, with average read depths of 10.2× (10.06× to 10.37×). On average, across all samples, the reads covered 99.26% of the genome (S1 Table).

### SNP calling

After alignment, we performed SNP calling using a Bayesian approach as implemented in the package SAMtools. Realigned regions were piped to SAMtools and reformatted into pileup files for SNP identification. Sequence variants from pileups were then condensed into a variant call format (VCF) file using BCFtools [27] (0.1.19). The genotype with maximum posterior probability was picked as the genotype for that locus.

The threshold of SNP calling was set to 20 for both base quality and mapping quality. SNPs were discarded based on the following conditions: (i) quality less than 20; (ii) those with too low (total depth < 2×20) or too high (total depth > 40×20) a depth (possibly bad assembly or repetitive regions); (ii) 5 bp around InDels; (iv) those occurring in a cluster (more than three SNPs with 10 bp); (v) failure in the exact test for Hardy-Weinberg equilibrium at P<0.001; or (vi) those with > 50% missing genotype data with the population.

### Phasing and linkage disequilibrium

The program Beagle [43] (version: r1196) was used to infer the haplotype phase and impute missing alleles with default parameters. Linkage disequilibrium (pairwise r^2^ statistic) was calculated using Haploview [44] (v4.2) software with the parameters ‘-dprime -maxDistance 1000 -minMAF 0.05 -hwcutoff 0.001 -missingCutoff 0.5 -minGeno 0.6’.

### Phylogenetic relationship and population structure analysis

To assess recent relationships between samples, we calculated pairwise estimates of Identity-By-State (IBS) scores [28]. We found no possible duplicate (IBS>0.9) that showed high pairwise genetic similarity with another sampled individual, indicating that these 20 individuals, as unrelated samples, can be used in the downstream analyses.

Neighbor-joining trees were constructed with PHYLIP (v3.696, http://evolution.genetics.washington.edu/phylip.html)) using the matrix of pairwise genetic distances (‘—cluster—distance-matrix’ of PLINK v1.07). The SmartPCA program from the EIGENSOFT [45] (v5.0.1) package was used to perform principle component analysis on the individuals that we sequenced with default parameters. A Tracy–Widom test was used to determine the significance level of the eigenvectors and no significant eigenvectors were found (S2 Table). In addition, ADMIXTURE [46] (v1.23, with default parameters) was used to infer the population substructure among the samples with number for population grouping parameter K set from 1 to 3.

### Association analysis using PLINK

To map the poll trait loci, we performed a case-control analysis between horned and polled yaks using the Dominant model with PLINK [28] (v1.07, with parameters ‘—model—model-dom—fisher’). This Dominant model assumes that an effect on phenotype is only seen if you have at least one copy of the minor allele. It categorizes individuals into two groups based on whether they have at least one minor allele A (either Aa or AA) or no copies of the minor allele (aa). Fisher’s exact test was used to analyze genotypic differences between the 10 horned and 10 polled samples.

### Population genetic statistics

The nucleotide diversity (π) and population-differentiation statistic (*F*_ST_) were calculated using VCFtools [47] (v0.1.12a) with a sliding window approach (50 kb window sliding in 10 kb steps).

*d*_xy_ was calculated as follows:

$$d_{xy}=\sum_{ij}x_{i}y_{i}d_{ij}$$

where, in two populations, x and y, d_ij_ measures the number of nucleotide differences between the i^th^ haplotype from x and the j^th^ haplotype from y.

XP-CLR values were calculated with default parameters using XP-CLR (v1.0).

### Re-annotating the associated region using RNA-seq data

To find out whether there were new genes or open reading frames within our GWAS loci, we mapped previous RNA-seq data of domestic yak [32] to scaffold526_1 of the yak genome (as described in the ‘Sequence quality checking and mapping’ section). Read depths of RNA from five tissues (brain, kidney, lung, liver and heart, each with two duplicates) were calculated using SAMtools (parameters of ‘samtools depth’) and visualized as shown in Fig 2D.

### Checking P_F_, P_C_ alleles and other structural variants

To check the existence of a P_F_ allele in yak, we mapped our sequencing reads to the BTA1 sequence (UMD3.1 genome build, downloaded from Ensembl release77, http://www.ensembl.org/). Sequencing depths for each sample around the genome region near the P_F_ allele were calculated using SAMtools. The relative depths of each sample were calculated and no P_F_ allele (i.e. 80 kb duplication) was found in polled yaks (S4 Fig).

Structural variants (insertions, deletions, tandem duplications and inversions) were discovered using Pindel [48] (v0.2.5a3), which uses a pattern growth approach to identify the breakpoints of these variants from paired-end short reads (with default parameters and ‘-c 1 -T 30 -l -k’). No P_C_ allele was found in polled yaks and no other structural variants were found associated with polled phenotypes.

## Supporting Information

S1 Fig Schematic of breeding practice for Datong yaks. Sexuality is indicated by a circle (cow) or a square (bull), genotypes are indicated by different colors (PP, orange; Pp, green; pp, blue).
(TIF)

S2 Fig Population structure plots with K = 1–3. The y axis quantifies the proportion of the individual’s genome from inferred ancestral populations, and the x axis shows the different populations. The CV error of each run is given in parentheses.
(TIF)

S3 Fig QQ-plot of GWAS *P* values. (TIF)

S4 Fig Relative depth of horned (blue) and polled (red) yaks around the P_F_ allele on BTA1. The green frame indicates the region of the P_F_ allele.
(TIF)

S5 Fig Genome-wide distribution of π_horned_ (a) and π_polled_ (b).
(TIF)

S6 Fig Genome-wide distribution of *F*_ST_. (TIF)

S1 Table Overview of sample information and sequencing statistics. (XLS)

S2 Table Tracy-Widom (*TW*) statistics and *P*-values for the first five eigenvalues in the PCA. No significant *P* values.
(XLS)

S3 Table Results of structural variants discovery. (XLS)
