# Supplementary figures and images for: Genome-wide Association Study Identifies Loci for the Polled Phenotype in Yak
Source: PLoS One. 2016 Jul 7;11(7):e0158642. doi: 10.1371/journal.pone.0158642 (PMC4936749; doi:10.1371/journal.pone.0158642)

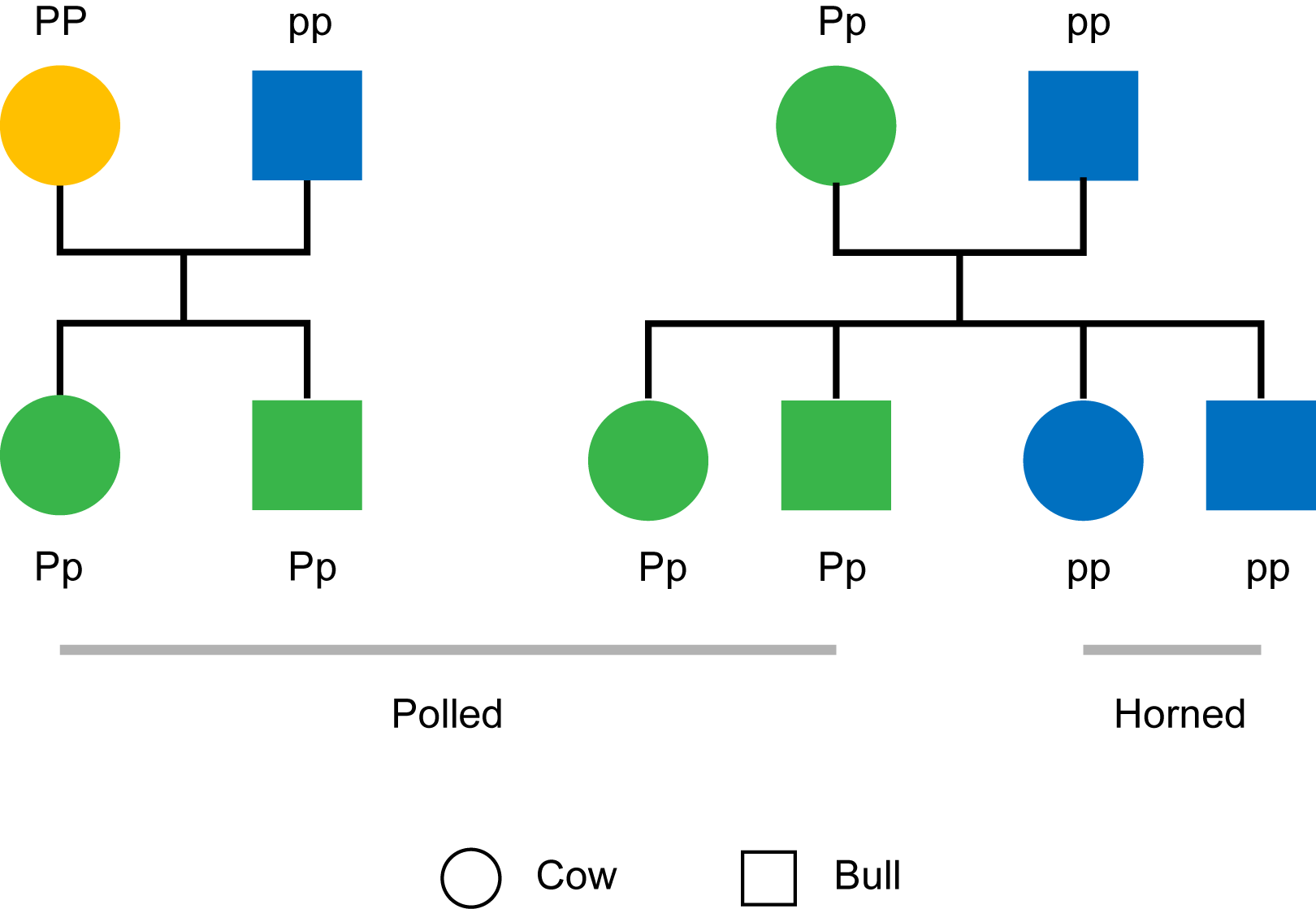

Supplement: S1 Fig — Sexuality is indicated by a circle (cow) or a square (bull), genotypes are indicated by different colors (PP, orange; Pp, green; pp, blue). (TIF) [file pone.0158642.s001.tif]

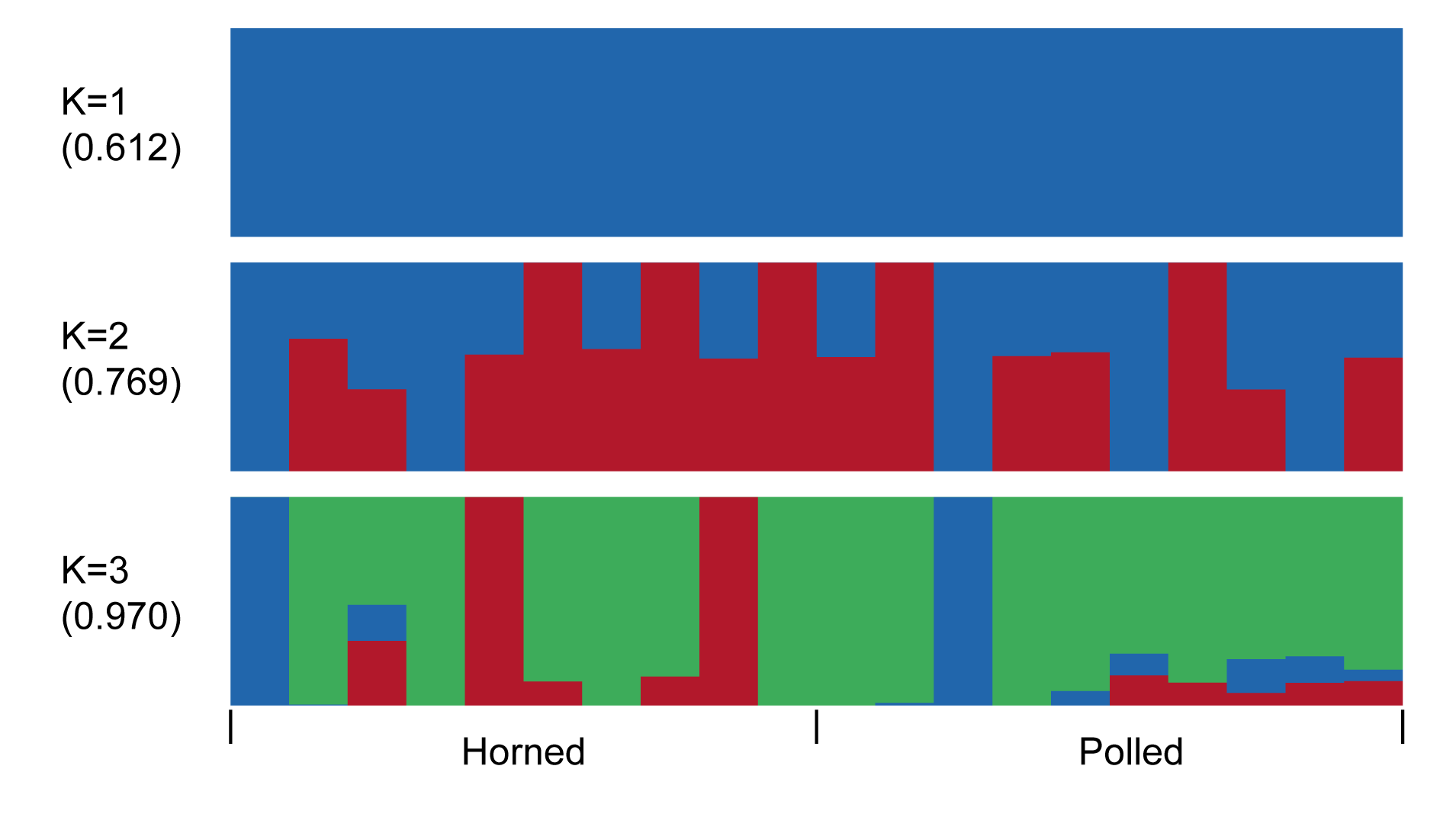

Supplement: S2 Fig — The y axis quantifies the proportion of the individual’s genome from inferred ancestral populations, and the x axis shows the different populations. The CV error of each run is given in parentheses. (TIF) [file pone.0158642.s002.tif]

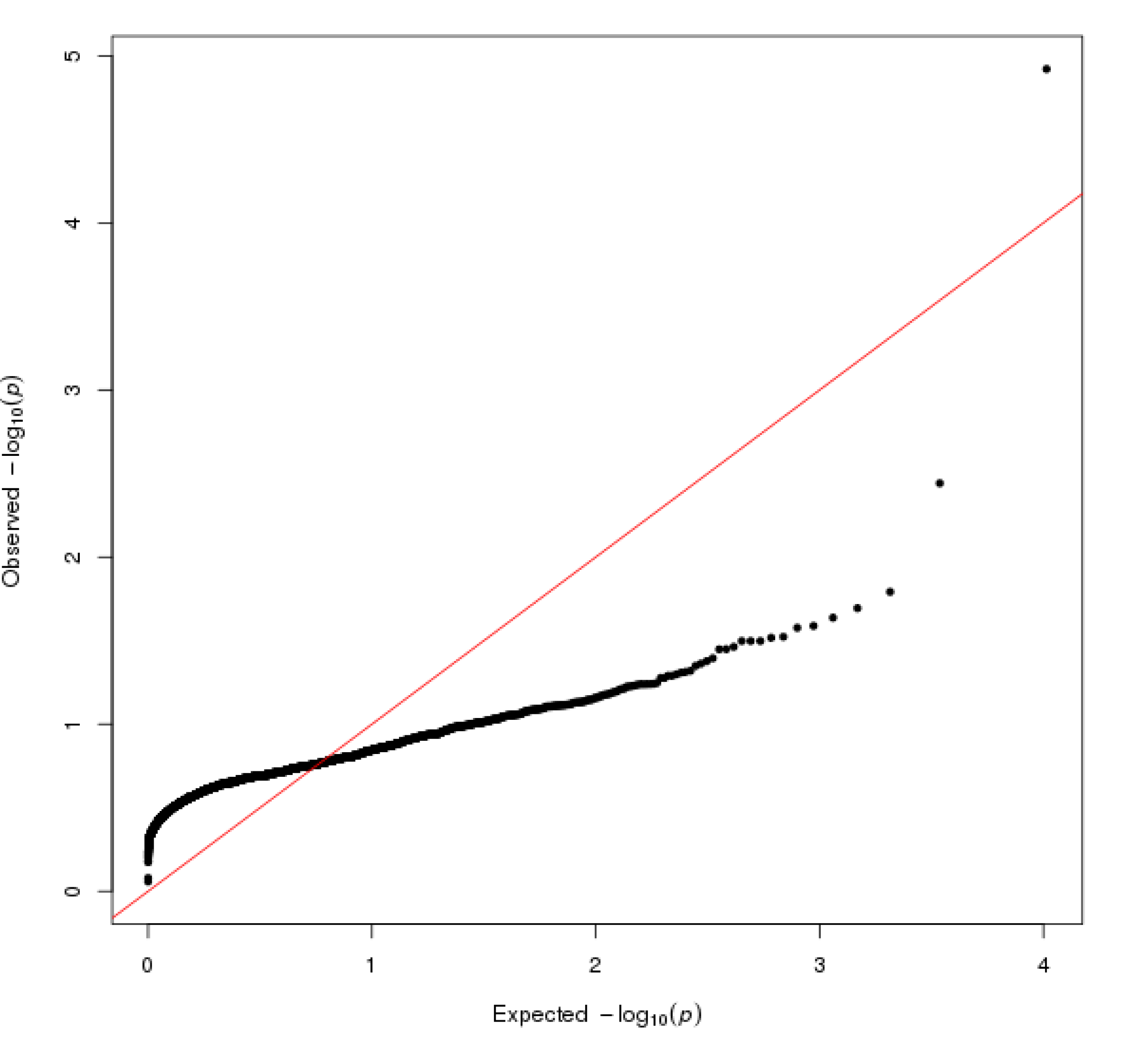

Supplement: S3 Fig — (TIF) [file pone.0158642.s003.tif]

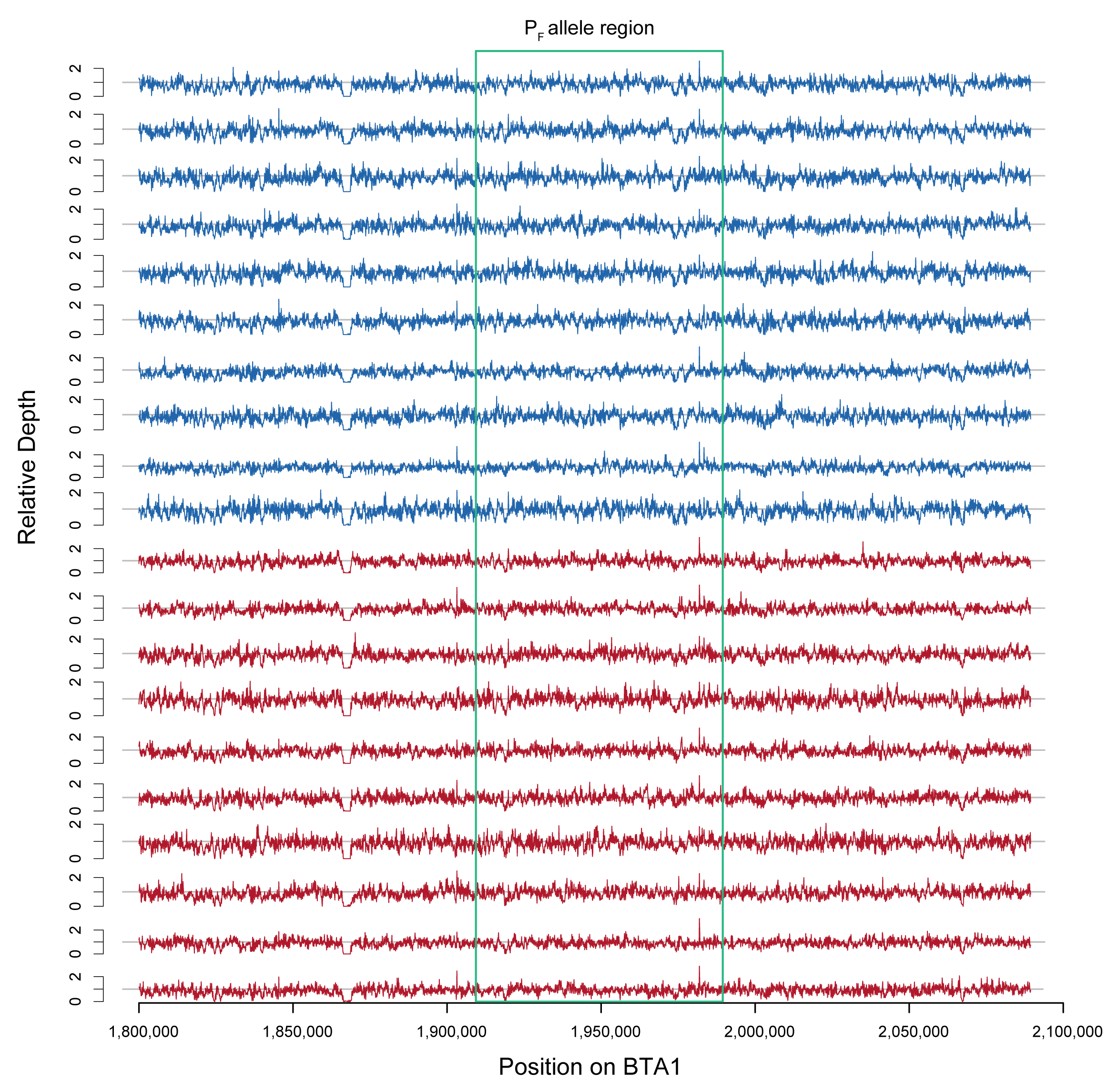

Supplement: S4 Fig — The green frame indicates the region of the PF allele. (TIF) [file pone.0158642.s004.tif]

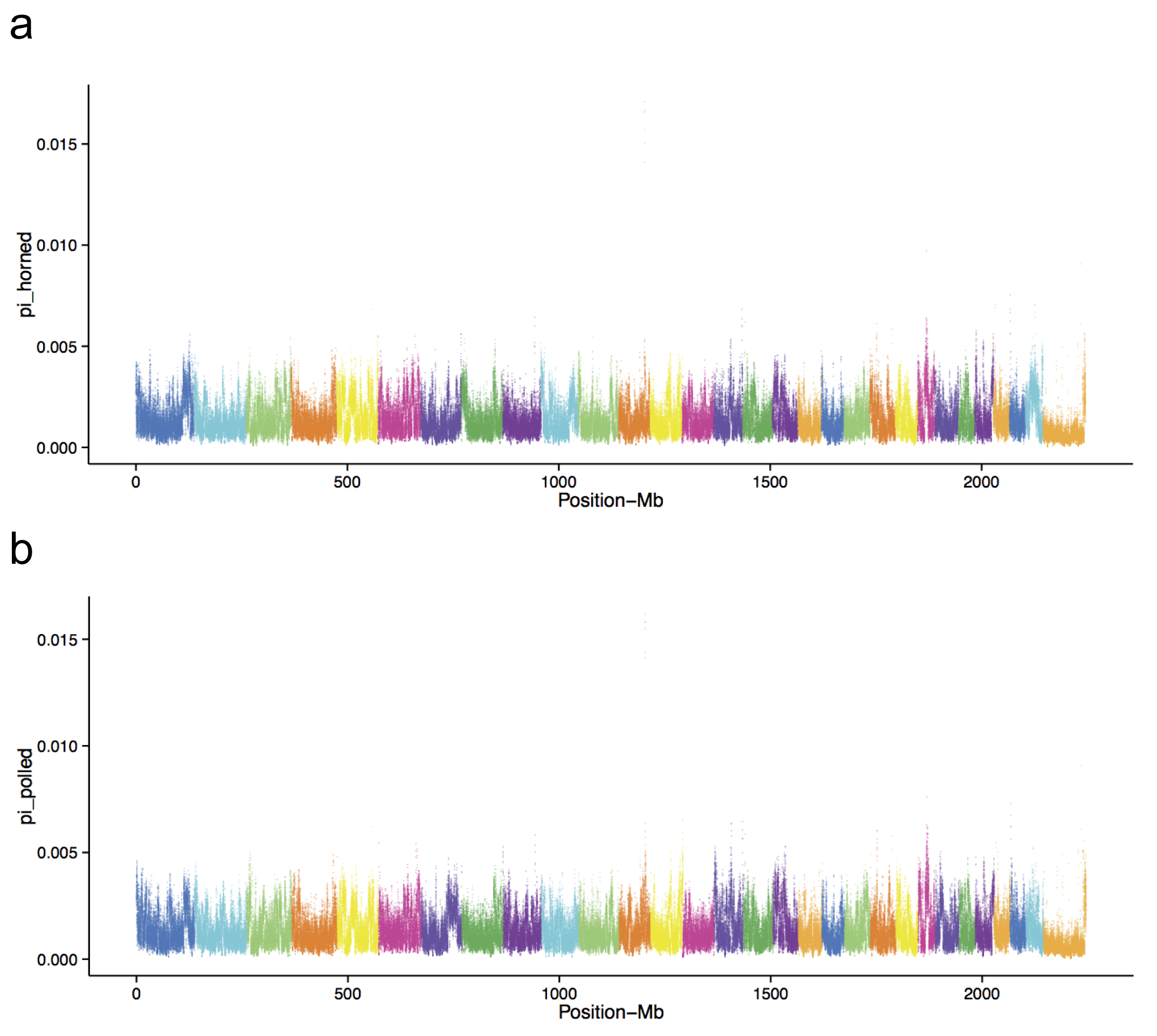

Supplement: S5 Fig — Genome-wide distribution of πhorned (a) and πpolled (b). (TIF) [file pone.0158642.s005.tif]

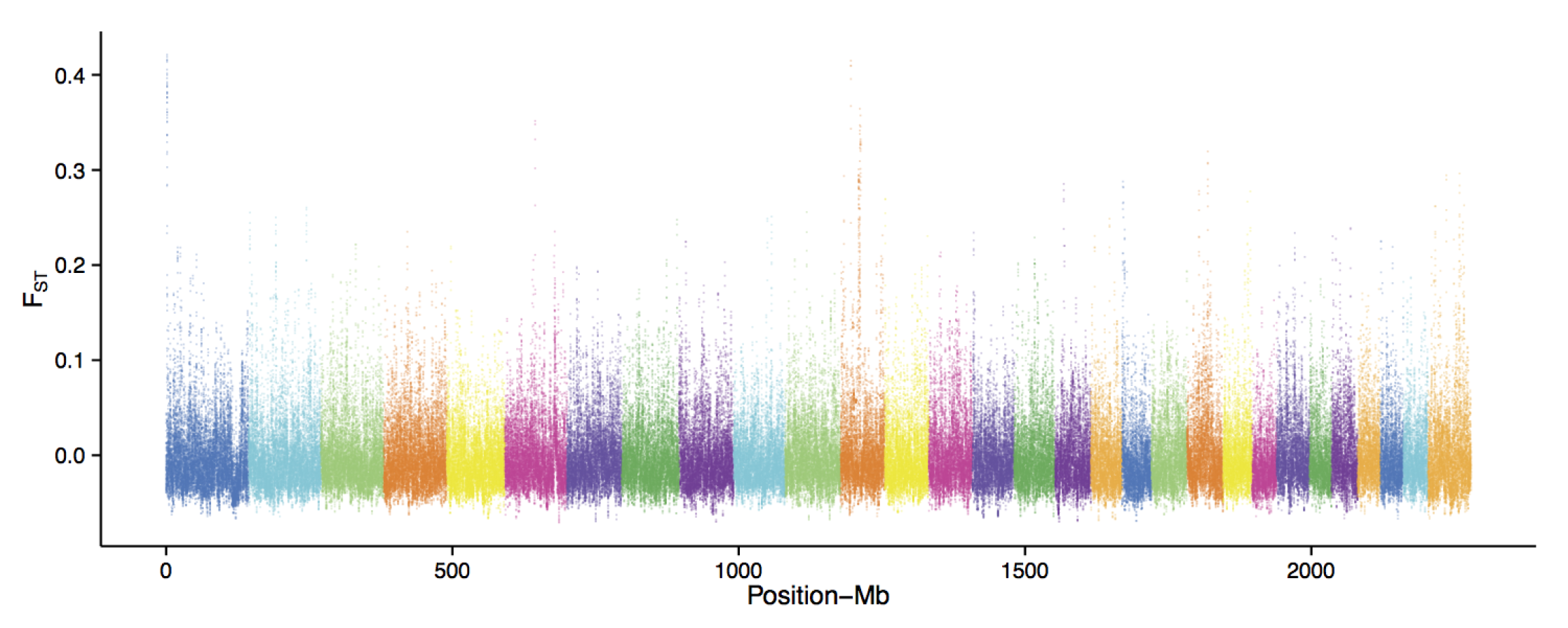

Supplement: S6 Fig — (TIF) [file pone.0158642.s006.tif]
